# Hospital physicians’ experiences with procalcitonin – implications for antimicrobial stewardship; a qualitative study

**DOI:** 10.1186/s12879-020-05246-6

**Published:** 2020-07-16

**Authors:** Ingrid Christensen, Jon Birger Haug, Dag Berild, Jørgen Vildershøj Bjørnholt, Lars-Petter Jelsness-Jørgensen

**Affiliations:** 1 Department of INFECTION Control, Østfold Hospital Trust, Kalnes, Norway; 2 Faculty of Medicine, University of Oslo, PhD Program Medicine and Health Sciences, Oslo, Norway; 3 Department of Infectious Diseases, Oslo University Hospital, Oslo, Norway; 4 Oslo University, Faculty of Medicine, Institute of Clinical Medicine, Oslo, Norway; 5 Department of Microbiology, Oslo University Hospital, Oslo, Norway; 6 Faculty of Health and Social Studies, Østfold University College, Fredrikstad, Norway; 7 Department of Science, Østfold Hospital Trust, Kalnes, Norway

**Keywords:** Antimicrobial stewardship, Procalcitonin, Hospital physicians, Semi-structured interviews, Qualitative study

## Abstract

**Background:**

Procalcitonin is an inflammatory biomarker that is sensitive for bacterial infections and a promising clinical decision aid in antimicrobial stewardship programs. However, there are few studies of physicians’ experiences concerning the use of PCT. The objective of this study was to investigate whether hospital physicians’ experience with procalcitonin after 18 months of use can inform the PCT implementation in antimicrobial stewardship programs.

**Materials/methods:**

We deployed a qualitative approach using semi-structured interviews with 14 hospital physicians who had experience with procalcitonin in clinical practice. Interviews were audio-taped, transcribed verbatim and analysed using thematic analysis.

**Results:**

Physicians reported a knowledge gap, which made them uncertain about the appropriate procalcitonin use, interpretation, and trustworthiness. Simultaneously, the physicians experienced procalcitonin as a useful clinical decision aid but emphasised that their clinical evaluation of the patient was the most important factor when deciding on antibiotic treatment.

**Conclusions:**

Procalcitonin was regarded a helpful clinical tool, but the physicians called for more knowledge about its appropriate uses. Active implementation of unambiguous procalcitonin algorithms and physician education may enhance the utility of the test as an antimicrobial stewardship adjunct.

## Introduction

Antimicrobial resistance (AMR) is a global health threat [1]. As one of several countermeasures, hospitals worldwide have established antimicrobial stewardship programs (ASPs) [2]. ASPs may be defined as “a coherent set of actions which promote using antimicrobials responsibly” [3]. One such potential action is to implement procalcitonin (PCT) as a clinical decision aid to improve antibiotic use. PCT is a biomarker that increases in response to bacterial rather than viral stimuli. Moreover, it rises rapidly after inflammatory stimuli (4–6 h) and has a short half-life of 24 h [4]. It has also been introduced in antibiotic stewardship as a decision aid to withhold antibiotic prescriptions and to reduce the duration of antibiotic treatment for various infections, without compromising patient safety [5, 6], in particular for patients with lower respiratory tract infections and sepsis [7, 8].

By 2017, most Norwegian hospitals had started to implement ASPs [9], and PCT was introduced in our hospital. However, in a recent survey of PCT routines in Norwegian hospitals, we found that 27 out of 28 hospitals (96%) used PCT, but only five (18%) had implemented PCT for ASP use, and none had done this proactively. Furthermore, only two institutions (7%) had systematically evaluated how PCT was used (unpublished data, J. B Haug, I. Christensen). Additionally, the adherence to PCT algorithms is generally low, of which the reasons are only partly understood [10–12]. There is consequently a need to explore physicians’ PCT use in more detail to improve the understanding of their behaviour and culture, which is recognised as essential in the development of sustainable ASPs [13–15]. This study aimed to investigate whether hospital physicians’ experience with PCT after 18 months of use can inform the PCT implementation in ASPs.

## Materials and methods

### Design and setting

This qualitative study, using semi-structured interviews, was conducted at Østfold Hospital Trust, a 380-bed acute care hospital in south-eastern Norway. As PCT was introduced in the hospital in April 2017, an antibiotic stewardship team had recently been established. At the time, this team mainly conducted surveillance of antibiotic use, revised guidelines and offered education on antibiotic use. ASP team members educated the hospital’s physicians in internal meetings on how to use the PCT assay, and a clinical algorithm was presented (Table 1) but insufficient resources were available to active follow up the physicians’ compliance. The hospital laboratory only referenced the standard electronic PCT cut-off value of 0.1 μg/L along with the PCT results.

**Table 1:** PCT algorithm recommended in internal meetings at the PCT introduction

**Intensive care unit (severe infections/sepsis)**	
PCT < 0.5 μg/	Antibiotics should be considered withdrawn
PCT ≥ 0.5 μg/L	In patients who have improved clinically, subsequent PCT analyses are recommended on days 3 and 5. A decrease of 80% of the initial value suggests that antibiotics are no longer needed.
**Respiratory tract infections** (community-acquired pneumonia and acute exacerbation of chronic obstructive pulmonary disease)	
PCT < 0.1 μg/L:	Strongly encourage against the continuation of antibiotics
PCT < 0.25 μg/L:	Encourage against the continuation of antibiotics
PCT 0.25–0.5 μg/L:	Recommendation for continuing antibiotics
PCT > 0.5 μg/L:	Strong recommendation for continuing antibiotics

Due to cost restraints, the use of PCT was restricted to intensive care, haematology, gastrointestinal surgery, infectious and pulmonary disease departments. In the following 18 months, the use of PCT was left to the physicians’ discretion. However, all physicians ordering PCT were prompted to fill out an electronic form in the patient’s medical records, asking for information concerning i) indication for PCT, ii) actions taken based on PCT results, and iii) their subjective view on the value of the test (Additional file 1). For the present study, the form was used to map the actual PCT usage in our hospital and give valuable information for the interview guide.

### Recruitment and data collection

Potential informants were identified from the laboratory database of PCT requesters. Included physicians (*n* = 14) had requested a mean of 20 PCT tests compared to a mean of four tests by the not-invited physicians (*n* = 219). An invitation to participate was not solely based on the number of PCT requests, but by a joint judgement by the authors IC and JBH of the candidates based on diversity in medical experience and speciality. The first author (IC) sent eligible physicians an email invitation to participate in the study which contained information about the aim and practical aspects of study participation, including the need to reserve 60–90 min of uninterrupted interview time.

An interview guide (Summarised in Table 2, full version in Additional file 2) was developed based on the literature [6, 10, 16, 17], and leveraged by the previously mentioned electronic form (Additional file 1). Three pilot interviews, with eligible physicians, were conducted to optimise the final interview-guide and technique, but were not included in the analysis.

**Table 2:** Summarised interview guide (full version in Additional file 2)

1. Can you please describe some of your experiences with the PCT-test?	
2. Can you recall the first time you used PCT?	
3. What are your expectations for the test?	
4. According to the medical literature, there is no firm consensus on PCT use. Could you describe how this matches your experiences?	
5. Have you received any education or guidance on PCT uses?	
6. Can you recall an episode when a PCT result was low, and you decided to start or continue antibiotic treatment?	

All interviews were performed by IC (a female junior doctor trained in qualitative methods, with clinical experience from surgery and oncology at the study hospital) and held in a quiet room at the hospital. The interview period lasted from November 2018 to February 2019. Following the 12th interview, no new themes were identified. To ensure saturation, we conducted two more interviews, resulting in a total of 14 interviews. The mean interview time was 52 min (23-74 min). Study participants included five from infectious diseases, three from oncology, one medical resident, and one from each of the following specialities: anaesthesiology, gastrointestinal surgery, gastroenterology, pulmonology, and haematology. Table 3 presents participant characteristics.

**Table 3:** Characteristics of study participants (*n* = 14)

**Age**	**(years)**
Median (IQR)	36.5 (14.5)
Range	29–66
**Hospital experience**	**(n)**
< 5 years	4
5–10 years	3
10–20 years	4
> 20 years	3
**Gender**	**(n)**
Female	9
Male	5

*IQR* Interquartile range

### Data management and analysis

Interviews were audio-recorded, transcribed verbatim, and de-identified (IC). Thematic analysis following the recommendations of Braun and Clarke was performed by IC and last co-author (LPJJ) [18]. IC scanned the transcripts for illustrative quotes. Scrutiny of our positions, presumptions, and their possible influences on the study process was continuously applied and written down in a project log (reflexivity) [19].

## Results

Two main themes were identified. Theme one, “knowledge gap”, consists of three subthemes: “unsure of use”, “unsure of interpretation”, and “trustworthiness”. Theme two, “diagnostic value”, consists of two subthemes: “supporting decisions” and “clinical evaluation most important.” Themes, subthemes, and representative quotes are presented in Table 4. After the quotes presented in the text, the respondent number and years of experience is given.

**Table 4:** Themes, subthemes and illustrative quotes

Example quotes (informant number, clinicians’ years of experience)	Subtheme	Theme
*My experience is that no one can actually say something certain about it (PCT). We use it and it guides us to some degree (…*) *but we don’t trust it 100% (R10, 16y)* *It (PCT) is possibly indicated in several cases, but I don’t know them, so I think more knowledge about it would be effective (R14, 1y)* *What you don’t use you don’t get good at; I see up to 100 CRP values every day so of course I can interpret CRP, while I encounter a PCT maybe only twice a week (R2, 18y)*	Unsure of use	**Knowledge gap**
*When I get the result, I have no clue what it means. Then I ask colleagues, and they just “no, we don’t quite know what it means, don’t know if we can trust it, don’t know whether it increases or decreases in certain infections” (R5, 4y)* *If someone had informed us how to interpret the (PCT) results for this patient group (cancer), it would of course have been a great help and I believe it would have made us a little more confident when using the test (R1, 12y)*	Unsure of interpretation	
*I am not sure I would trust PCT in all diagnoses. The other day in geriatrics there was a lady with a HUGE intra-abdominal abscess, she had a PCT which was 0.25, which isn’t much (R11, 22y)* *I’ve gotten surprised once in a while when I’ve used PCT* e.g. *on patients receiving immunotherapy; they are admitted with suspected infection, but it is actually adverse effects of the immunotherapy and PCT turns out really high, which is very confusing as there are no bacteria involved. (R5, 4y)*	Trustworthiness	
*If I document that the patient has (increased) CRP but the PCT is only 0.27, he has no fever or other clinical signs of infection, I can quit antibiotics, − it helps me to be legally sound with regard to that decision (R8, 23y)* *Very many patients get antibiotics “just in case”, as we say, but after we got PCT; it is absolutely a decision aid that helps us being “brave enough” to stop antibiotics or to not start antibiotics. (R4, 25y)* *I think it is most useful to give backing in a decision. (R10, 16y)*	Support decisions	**Diagnostic value**
*She had a CRP at 300 and high fever and all sepsis criteria, she also had an increased PCT, but it didn’t matter, she would have gotten antibiotics either way. (R13, 4y)* *If I am quite convinced it is not an infection, but request a PCT and it turns out positive, I dismiss it and say like “no, I don’t think it is an infection’ and base my decision on the clinical picture (R5, 4y)* *The clinical picture was already enough for us to continue antibiotics (despite of low PCT), we would never stop antibiotics on that clinical appearance (septic cancer patient) (R6, 4y)*	Clinical evaluation most important	

### Theme one: knowledge gap

#### Unsure of use

Uncertainty was related to the actual *use* of the test, an aspect that was reported both directly by the physicians and through their descriptions of clinical situations. In particular, uncertainty was expressed as to which indications it was appropriate to use PCT: “*It (PCT) is not to be used for all diagnoses, I don’t quite remember ( …*) *I would like more experience; when is it indicated to use it?” (R11, 22y).* The physicians highlighted that uncertainty of use was a driver for not trusting and using the test more: *“If we knew how to use it (PCT) correctly and had more competence on its use ( …*) *then it could be more helpful” (R13, 4y).* Moreover, the test was infrequently used, so obtaining information about the test from the scientific literature was not prioritised in a busy clinical practice. The physicians that had consulted the literature were still unsure due to diverse guidelines and lack of familiarity with the test, advocating the need for more experience: *“I don’t have enough experience with it, to verify it; we sort of have to test it, so I think time will show” (R1, 12y).*

#### Unsure of interpretation

Another area of uncertainty was on *how* to interpret the test results. If clear-cut guidelines had been available, this could have facilitated faster and more targeted decisions: *“If we had straightforward guidelines ( …*) *and knew that when a patient with PCT above this or that level most likely has sepsis, then it (PCT) would probably be pretty helpful”* (R13, 4y). When physicians encountered PCT levels referred to as “median,” “in-between” or “borderline values”, they fell short in interpreting the results: *“I don’t quite have a sense of it yet; what is actually really high, what is low, and what is in between (values)?”* (R8, 23y). In fact, when left with “*borderline values,*” i.e. values which the physicians were unsure of, they usually chose to give antibiotic treatment “just in case.”

#### Trustworthiness

Several physicians reported experiences where the PCT results had “scared,” “failed,” or “disappointed” them, which led to uncertainty about the tests’ trustworthiness. E.g., when the PCT result was surprisingly deviant from their expectations based on the clinical picture: “*We recently had a patient where PCT increased to very high levels, but we did not have any other indicators for infection ( …*); *therefore, we used the result to observe that PCT is not the answer to everything” (R4, 25y)*. Some reported frightening experiences;” *it was a close call that the patient survived; negative PCT and pneumococcus in the aorta, but there was no sepsis (…). THAT one scared us; it was completely negative PCT” (R7, 13y).* Such experiences led to a lack of confidence in the test and, in some cases, physicians had stopped using it. The majority of physicians, however, had continued to use the test but applied it more cautiously.

### Theme two: diagnostic value

#### Supporting decisions

Physicians found PCT supportive for clinical decision-making involving infections, in particular when they referred to the results as either “very high” or “low,” as opposed to the “middle values.” Physicians framed this support in various ways, for instance informant 4 (25y) emphasised that PCT made him *“brave enough to stop antibiotics”,* while others highlighted that it provided *“legal support”* (R8, 23y), or *“put more flesh on the bones”* (R13, 4y). They especially appreciated when the test supported their premade decision: *“The clinical findings may not be convincing with regard to infection, so we order a PCT; if it is negative, we kind of get more support to quit antibiotics”* (R14, 1y). Several of the physicians had experienced PCT as an additional tool to traditional infection markers, such as C-reactive protein (CRP) and leukocytes.

The physicians viewed PCT as particularly useful in cancer patients. In these cases, clinical decisions were often perceived as challenging since fever, impaired general condition or increased CRP could relate to cancer itself or an infection: *“Increased CRP – it does not necessarily mean an infection in cancer patients; in these cases, PCT is a good tool to obtain an overall picture ( …*)” (R6, 4y).

An unexpected PCT value also guided the physicians to think more broadly and sometimes accelerated the diagnostic process. For example, looking for rheumatologically or malignant disorders when the result was lower than expected, or for infection when the result was higher than expected: *“In one patient, we were in some doubt about a possible rheumatic condition ( …*); *then, we requested a PCT which turned out to be 17, which led us to look more thoroughly for an infection”* (R11, 22).

#### Clinical evaluation most important

Although PCT was viewed as a contribution to the diagnostic toolbox, it was not a standalone test. Some had felt great enthusiasm when it was introduced, but as they gained experience, they realised that PCT could not replace clinical judgement. The patient’s clinical appearance remained the most important basis for decisions: “*I do not feel PCT is the answer to everything, we have to look at the CLINICAL APPEARANCE; you know, and what kind of disease is it? Where is the infection? Therefore, I do not base all my decisions on it”* (R11, 22). One informant, however, reported on an episode in which a high PCT overruled the clinical picture and led to the prolonged use of antibiotics: *“We expected the PCT to be low based on the clinical picture, but it was high, so we continued with antibiotics, even though the clinical picture was not convincing of infection”* (R14, 1y).

## Discussion

This study has shown that physicians experience a knowledge gap related to the use of PCT, which acts as an important barrier to optimal use. The physicians, however, perceived PCT to be a helpful tool in clinical decision making, but the most crucial factor for antibiotic prescription remained their clinical assessment of the patient.

A lack of clear guidelines on how to use PCT and the relatively short-term experience with PCT were factors the physicians pointed out to, a least partly, explain the knowledge gap. This gap barricaded for optimal use as it resulted in the use of PCT in clinical issues in which it had not been validated. For instance, the physicians questioned the trustworthiness of PCT when they used the test for focal infections, although such use is not recommended in the literature [6]. Moreover, the physicians found PCT helpful to decide whether or not to initiate antibiotics and in differentiating infections from, e.g., cancer and rheumatic disorders, neither of which are recommended by the literature [20]. To date, respiratory tract infections (RTI) and sepsis are the only diagnoses in which meta-studies confirm the benefits of routine PCT use. In RTI, PCT is recommended to guide the decision to withhold or stop antibiotics and, for sepsis, to guide the discontinuation of antibiotics [21]. Moreover, despite the literature stating that PCT cannot be trusted in all diagnoses, some physicians had lost faith in the test due to a mismatch between the PCT results and their clinical assessment. The varying recommendations across studies (e.g., indications, timing, and cut-off values) may furthermore complicate rather than clarify the optimal use of PCT [22]. Even experienced physicians requested “a clear manual.” Recently, two consensus reports on PCT-guided antibiotic therapy have been published and provide updated recommendations [23, 24]. Both of these reports recommend individualising decisions by evaluating PCT cut-off values together with disease severity, setting (hospital department), clinical evaluation, and other test findings (e.g., microbiological). The recommendations are comprehensive and thus not straight forward from a full-time clinicians’ perspective.

Another explanatory factor of the knowledge gap, and thus the suboptimal PCT use, is that there was no ongoing PCT implementation at the time of this study. Recent real-world studies have shown diverging results that underline the role of active implementation. One study, in which there had been no PCT implementation, found *increased* days of antibiotics in the patients whom the physicians had used PCT [25]. On the contrary, other studies, in which PCT implementation was a part of an ASP, found significantly *decreased* antibiotic days of therapy [26, 27]. These findings, combined with our study physicians’ call for more explicit guidance, highlight a need for enforced PCT implementation with clear-cut instructions.

Our finding of perceived usefulness of PCT for other indications than recommended in guidelines underscores a need for education on the current evidence, but also the need for further studies into the potential role of PCT in other diagnoses, such as cancer [6].

Systematic reviews on the determinants of antibiotic prescription commonly report that physicians prescribe excess antibiotics due to anxiety about overlooking severe infections [28–30]. In our study, several informants emphasised that PCT could limit such anxiety and thus promote a more rational antibiotic prescription. The informants experienced that PCT could not replace other tests or clinical judgment, but they still valued it as a diagnostic adjunct.

Adherence to PCT algorithms is commonly low, but explanatory factors remain relatively unknown [11, 31]. Given the well-documented reduction in inappropriate antibiotic use by adherence to PCT algorithms, optimising adherence should be prioritised [7, 8]. In the current study, two factors were identified that might provide explanatory insight into low adherence. First, uncertainty about interpretation may result in the prescription of antibiotics “just in case,” thus overruling PCT algorithms. Secondly, clinical judgement is regarded as a more critical factor for an antibiotic decision than the PCT value. Both elements align with studies where physicians, when feeling a clinical uncertainty, have prescribed antibiotics despite the PCT value being low [31, 32]. However, studies have failed to show any independent association between patient clinical severity and PCT algorithm compliance [10, 11]. Consequently, low adherence cannot be explained solely by the severity of patients’ clinical condition, but may instead be driven by the identified knowledge gap of when and how to use PCT. A logical consequence may be that ASP teams should increase their attention towards a targeted education on when PCT is indicated and when it is not. Also, the team should allocate time to fulfil their paramount role in active guidance and follow-up of physicians during implementation.

While the qualitative design of our study has enabled us to pinpoint areas that need more focus and potentially can optimise ASP practices, the study also has some limitations. First, we did our research at a single centre, and other perspectives might have been identified in different settings, e.g. in hospitals having used PCT for a prolonged period or with physicians more thoroughly trained in PCT use than ours. However, for many hospitals where PCT is a new diagnostic commodity, we think the present study is relevant. At least in a Norwegian context, our findings are expected to make a difference as judged by an aforementioned national survey of PCT use in hospitals. Although many Norwegian centres use PCT, an evaluation of its clinical usefulness was seldom performed. Also, in most hospitals, utilisation of PCT for clinical diagnostic indications seems to be far more prevalent than for antibiotic stewardship purposes. We assume that a similar situation may exist even in other countries.

Secondly, the primary investigators (ICs) familiarity with some of the informants may have affected their responses. However, a deliberate process of reflexivity was deployed to limit the influence of IC’s position.

## Conclusion

The physicians valued PCT as a decision aid in antibiotic prescribing. Still, uncertainty about the test acted as a barrier for optimal use, which may be remedied by straight forward PCT guidelines and rigorous education and support of the physicians in future antimicrobial stewardship efforts.

## Supplementary information

**Additional file 1.** Additional file 1_Point_of_care_questionnaire_PCT_use: Point of care questionnaire of the uses and consequences of PCT use.

**Additional file 2.**Additional file 2_Interview_guide: Interview guide.

## Data Availability

The datasets supporting the conclusions of this article are available from the corresponding author on reasonable request. They are not deposited in a public repository as the transcripts could potentially reveal identifiable information.

## Abbreviations

AMR: Antimicrobial resistance
ASP: Antimicrobial stewardship program
PCT: Procalcitonin
RTI: Respiratory tract infections
