# Supplementary material for: Hospital physicians’ experiences with procalcitonin – implications for antimicrobial stewardship; a qualitative study
Source: BMC Infect Dis. 2020 Jul 16;20:515. doi: 10.1186/s12879-020-05246-6 (PMC7364625; doi:10.1186/s12879-020-05246-6)
Supplement: Supplementary file 1 — Additional file 1. Additional file 1_Point_of_care_questionnaire_PCT_use: Point of care questionnaire of the uses and consequences of PCT use. [file 12879_2020_5246_MOESM1_ESM.pdf]

Point of care questionnaire of uses and consequences of procalcitonin (PCT) use

SINGLE-measurement [ ] SERIAL-measurement [ ] Total number of measurements: …….

(PART1) INDICATIONS

A: ***The patients is treated with antibiotics*** and ***stopping*** is considered:

- ***Serious infection/sepsis, Adult intensive*** (not COPD exacerbation) [ ]
  Assumed diagnosis: ……….. Comment: …..

- ***Community-acquired pneumonia*** 1) PCT first 24hours [ ] - 2) PCT at day 5-7 [ ] (**X** for yes)
  Comment: …………

- ***COPD exacerbation*** 1) PCT first 24hours [ ] - 2) PCT at day 3-5 [ ] (**X** for yes)
  Comment: …………

- **Other infection**: [ ] Diagnosis: ………..
  Comment: ………….

B: ***Influensa***

- **Suspected flu** 1) PCT first 24 hours [ ] - 2) PCT at day 5 [ ] (**X** for yes)

  Comment: ………..

C: ***Another differential diagnosis:*** [ ] (**X** for yes)
Short description of the diagnosis: ……………..

(PART 2) FOLLOW UP – *PCT result evaluated* (date and time): ……..

Did the PCT-analysis affect your clinical decision? Yes [ ] No [ ]

If YES, how? …..

If NO, why not? …..

On a 1-10 scale, to what degree do you think the PCT result affected your decision? …..
