# Supplementary material for: Hospital physicians’ experiences with procalcitonin – implications for antimicrobial stewardship; a qualitative study
Source: BMC Infect Dis. 2020 Jul 16;20:515. doi: 10.1186/s12879-020-05246-6 (PMC7364625; doi:10.1186/s12879-020-05246-6)
Supplement: Supplementary file 2 — Additional file 2.Additional file 2_Interview_guide: Interview guide. [file 12879_2020_5246_MOESM2_ESM.pdf]

Interview guide

1. Can you please describe some of your experiences with the PCT-test?
   Probes: please try to recall some recent episodes when you used the test and describe it in detail.

2. Can you recall the first time you used PCT?
   Probes: has the use changed? If YES: how?

3. What are your expectations for the test?
   Probes: do you find it useful? How do you think PCT affects clinical practice?

4. According to the medical literature, there is no firm consensus on PCT use. Could you describe how this matches your experiences?

5. Have you received any education or guidance on PCT uses?
   Probes: If YES, can you please describe it? If NO, can you please explain how you would like such guidance for it to be of the most value?

6. Can you recall an episode when a PCT result was low, and you decided to start or continue antibiotic treatment?
   Probes: if YES, can you describe the evaluations you made to make the decision? Were there any factors that could have affected you not to continue antibiotics?
